# Hyeropic shift after LASIK induced Diffuse lamellar keratitis

**DOI:** 10.1186/1471-2415-6-19

**Published:** 2006-04-28

**Authors:** Tanuj Dada, Mayank S Pangtey, Namrata Sharma, Rasik B Vajpayee, Vishal Jhanji, Harinder Singh Sethi

**Affiliations:** 1 Dr Rajendran Prasad Center for Ophtahlmic Sciences, All India Institute of Medical Science, NewDelhi-110029, India

## Abstract

**Background:**

Diffuse lamellar keratitis (DLK) is a relatively new syndrome that is increasingly being reported after LASIK. We have observed that a hyperopic shift may be associated with the occurrence of this diffuse lamellar keratitis.

**Case presentation:**

A 26 year old man developed bilateral diffuse lamellar keratitis (DLK) following myopic LASIK. The residual refractive error was +0.5D OD and +0.25D OS at the end of the first week. The sterile infiltrates resolved over a period of 4–6 weeks on topical steroid therapy. A progressive hyperopic shift was noted in the right eye with an error +4.25Dsph/+0.25Dcyl 20 at the final follow up 6 months post surgery.

**Conclusion:**

Diffuse lamellar keratitis after LASIK may be associated with a significant hyperopic shift.

## Background

Laser in situ keratomileusis (LASIK) has become the refractive procedure of choice for myopia [1,2] and hyperopia [3] due to its inherent advantages of a quick recovery time, preservation of bowman's layer, and a clearer more organized anterior stroma [4]. Diffuse lamellar keratitis (DLK) is a relatively new syndrome that is increasingly being reported after LASIK [5]. It is characterized by the appearance of diffuse, multi focal, polymorphonuclear infiltration in the flap interface, between 1–5 days after the surgery. The exact etiology of this noninfectious syndrome is still obscure. We have observed that a hyperopic shift may be associated with the occurrence of this diffuse lamellar keratitis.

## Case presentation

A 26 year old patient underwent LASIK for myopia of - 10.0 Dsph OD and -10.25 Dsph/-1.50 Dcyl @160 OS. The informed consent was obtained from the patient. He had not undergone previous keratorefractive surgery and there was no evidence of any other ocular or systemic disease. The best-corrected visual acuity was 20/20 in both eyes. The preoperative keratometry was 43.1 @ 179 degree and 44.1@ 89 degree (Orbscan) OD and 43.7@ 160 degree & 44.5@ 70 degree OS. The preoperative central corneal thickness (optical pachymetry, Orbscan) was 511 μOD and 516 μOS respectively.

The surgery was performed using the Chiron Technolas 217 eximer laser machine and the hansatome was used to create a 9.5 mm diameter flap, 160 μ in thickness. The optic zone diameter was kept at 5 mm for both eyes. In the right eye 130 μ of the stroma was ablated and in the left eye 148 μ was ablated.

The patient was noted to have a diffuse infiltration confined to the flap interface, with multiple foci of increased density in the center of the flap on the first postoperative day and was started on one hourly, 1% Prednisolone acetate eye drops. The uncorrected visual acuity was 20/200 OD and 20/400 OS. The patient was advised to undergo irrigation under the flap but refused any further surgical intervention. The infiltrates resolved gradually on topical steroid therapy over a period of 4 weeks. The best corrected visual acuity improved to 20/30 in both eyes but the end result was a significant hyperopic error. The visual acuity, refraction, corneal pachymetry and corneal topography were recorded preoperatively and during the follow up visits at 1 week, 1 month, 3 months and 6 months (Table 1).

**Table 1:** Pre & Postoperative visual acuity, Refraction, Pachymetry & Corneal Topography

			**BCVA**	**Refraction**	**Pachymetry(μ)**	**Corneal Topography**
**Pre Operative**	**Right Eye**		20/20	-10.0Dsph	511	44.1@89°/43.1@179°
	**Left Eye**		20/20	-10.25Dsph/-2.5Dcyl@165°	506	44.6@70°/43.7@160°
**Post Operative**	**1**^st ^**Week**	Right	20/80	+0.50Dsph	289	41@79°/39.8@169°
		Left	20/60	+0.25Dsph	325	41.7@67°/39.6@157°
	**1**^st ^**Month**	Right	20/30	+0.75Dsph	314	40.9@73°/39.9@163°
		Left	20/30	+0.25Dsph	333	41.4@70°/39.7@160°
	**3**^rd ^**Month**	Right	20/30	+4.0Dsph/+1.75Dcyl@20°	323	41.1@84°/39.3@174°
		Left	20/30	+0.75Dcyl@180°	347	41.2@76°/39.7@166°
	**6**^th ^**Month**	Right	20/30	+4.25Dsph/+0.75@20°	361	41.1@90°/39.7@0°
		Left	20/30	+0.24Dcyl@155°	343	41.2@75°/39.7@165°

## Discussion

Diffuse Lamellar Keratitis (DLK) or the Sands of Sahara syndrome is a well recognized complication of lamellar surgery. It is a self perpetuating sterile inflammation of the cornea following any intervention where a lamellar incision has created an interface through stromal tissue. The incidence has been reported to be 0.2% but is believed to be much higher^6^. Various factors that have been implicated include toxic insult due to organic esters and lubricants from lubricants and machine oil, particulate matter on the microkeratome blade, release of endotoxin from bacterial growth on the reusable instruments after autoclaving, traumatic insult from microkeratome, hemorrhage from a micropannus, heating effect of laser on the cornea, meibomian gland secretion, povidone iodine, debris from gloves, absorbent sponges and particles from an adhesive catheter dressing used as a drape [7-12]. The present case represents a stage 3 DLK, which may be seen on day 1, and is characterized by a severe inflammation with a decrease in visual acuity and must be managed by manually lifting the flap and irrigating under it. [15,16]

The unique feature in this case was the excessive hyperopic shift after the episode of lamellar keratitis. Previously Smith et al [5] reported 11 cases of diffuse lamellar keratitis of which 3 cases developed hyperopic shift. Of these three cases a 44 years old man who underwent LASIK with astigmatic keratotomy developed the highest shift of +0.75 Dsph/+1.25 Dcyl @ 86 degree. Peters et al [13] has reported 18 eyes with DLK which had a mean postoperative refractive error of -0.07 ± 0.48D. in another group of same study, 7 eyes developed DLK with a mean refractive error of +0.04 ± 0.32D.

Possible reasons for this shift could be overcorrection, scarring of the stroma with irregular astigmatism and flattening, stromal tissue loss due to destructive neutrophilic enzymes [15,16]and deposition of material with an abnormal refractive index. In the first week the refractive error was +0.5D sph in the right eye and +0.25 Dsph in left eye (Table 1). So the excessive hyperopic shift due to an overcorrection is ruled out. Corneal edema could be the cause of a transient hyperopia but the likely effect would resolve over a fortnight. Tissue loss cannot be the implicating factor because there was an increase in the central corneal pachymetry on subsequent follow up (Table 1). If the inflammation is not controlled at an early stage, permanent stromal scarring and irregular astigmatism could lead to a hyperopic error [15,16].

Another hypothesis for the hyperopic shift is the hyperactive neutrophilic response, which along with the injured keratocytes degranulates, releasing collagenase as well as protease enzymes [14], and lead to excessive enzymatic thinning of stroma. This may act as a stimulus for compensatory stromal synthesis by the keratocytes and deposition of tissue with an abnormal refractive index, a factor which can only be proven by histochemical/electron microscopic analysis of the tissue.

Early intervention after the onset of DLK with copious irrigation of the stromal bed after lifting the flap may limit the inflammatory debris and help to prevent the occurrence of this complication although all steps should be taken for a primary prevention of this syndrome. [15,16]

## Conclusion

Diffuse lamellar keratitis after Laser in situ keratomileusis may be associated with a hyperopic shift.

## Competing interests

The author(s) declare that they have no competing interests.

## Authors' contributions

TD: Performed the surgery and prepared a manuscript

MP: Performed the literature search and helped in the documentation of the case

RBV: Helped in making the clinical diagnosis and management of the case

NS: Helped in making the clinical diagnosis and management of the case

VJ: Helped in maintaining the follow up and data of the case

HSS: Helped in Preparation and submission of the manuscript

All the authors read and approved the final script.

## Pre-publication history

The pre-publication history for this paper can be accessed here:



## References

[B1] Pallikaris IG, Siganos DS (1994). Excimer laser in situ keratomileusis and photorefractive keratectomy for correction of high myopia. J Cataract Refact Surg.

[B2] Knorz MC, Liermann A, Seiberth V (1996). Laser in situ keratomileusis to correct myopia of 6.00 to 29.00 dioptres. J Refact Surg.

[B3] Buzard KA, Fundingsland BR (1999). Excimer Laser assisted in situ keratomileusis for hyperopia. J Cataract Refract Surg.

[B4] Amm M, Werzel W, Winter M (1996). Histopathological comparision of photorefractive keratectomy and laser in situ keratomileusis in rabbits. J Refract Surg.

[B5] Smith RJ, Maloney RK (1998). Diffuse Lamellar Keratitis; A new syndrome in lamellar refractive surgery. Ophthalmology.

[B6] Machat JJ, Machat JJ, Slade SG, Probst L, Lasik complications (1999). The Art of LASIK.

[B7] Kaufman SC, Maitchouk DY, Chiou AGY, Beuerman W (1998). Interface inflammation after laser in situ keratomileusis. Sands of Sahara syndrome. J Cataract Refract Surg.

[B8] Schultz CL, Morck DW, McKay SG, Olsen ME, Buret A (1997). Lipopolysaccharide induced acute red eye and corneal ulcers. Exp Eye Res.

[B9] Costerton JW, Lewandowski Z, Caldwell DE, Korber DR, Lappin-Scott HM (1995). Microbial biofilms. Annu Rev Microbiol.

[B10] MacRae S, Macaluso DC, Rich LF (1999). Sterile interface keratitis associated with micropannus hemorrhage after laser in situ keratomileusis. J Cataract Refract Surg.

[B11] Hirst LW, Vandelleur KW (1998). Laser in situ keratomileusis interface deposits. J Refract Surg.

[B12] Lachance A, Tremblay M (1998). The sands of Sahara. Can J Ophthalmol.

[B13] Peters NT, Lingua RW, Kim CH (1999). Topical intrastromal steroid during laser in situ keratomileusis to retard interface keratitis. J Cataract Refract Surg.

[B14] Apple DJ, Rabb MF (1991). Ocular Pathology: Clinical applications and self assessment.

[B15] Macaluso DC, MacRae SM, Gimbel HV, Anderson Penno EE (2001). Diffuse Lamellar Keratitis. LASIK complications : Prevention and management.

[B16] Linebarger EJ, Hardten DR, Lindstrom RL (2000). Diffuse lamellar keratitis: diagnosis and management. J Cataract Refract Surg.

